# Base excess is associated with the risk of all-cause mortality in critically ill patients with acute myocardial infarction

**DOI:** 10.3389/fcvm.2022.942485

**Published:** 2022-08-09

**Authors:** Chaodi Luo, Zhenzhen Duan, Tingting Zheng, Qian Li, Danni Wang, Boxiang Wang, Pengjie Gao, Dan Han, Gang Tian

**Affiliations:** ^1^Department of Cardiology, First Affiliated Hospital of Xi’an Jiaotong University, Xi’an, China; ^2^Department of Peripheral Vascular Diseases, Honghui Hospital of Xi’an Jiaotong University, Xi’an, China; ^3^Department of Cardiovascular Surgery, First Affiliated Hospital of Xi’an Jiaotong University, Xi’an, China

**Keywords:** base excess, blood gas analysis, acute myocardial infarction, prognosis, all-cause mortality

## Abstract

**Background:**

Base excess (BE) represents an increase or decrease of alkali reserves in plasma to diagnose acid-base disorders, independent of respiratory factors. Current findings about the prognostic value of BE on mortality of patients with acute myocardial infarction (AMI) are still unclear. The purpose of this study was to explore the prognostic significance of BE for short-term all-cause mortality in patients with AMI.

**Methods:**

A total of 2,465 patients diagnosed with AMI in the intensive care unit from the Medical Information Mart for Intensive Care III (MIMIC-III) database were included in our study, and we explored the association of BE with 28-day and 90-day all-cause mortality using Cox regression analysis. We also used restricted cubic splines (RCS) to evaluate the relationship between BE and hazard ratio (HR). The primary outcomes were 28-day and 90-day all-cause mortality.

**Results:**

When stratified according to quantiles, low BE levels at admission were strongly associated with higher 28-day and 90-day all-cause mortality. Multivariable Cox proportional hazard models revealed that low BE was an independent risk factor of 28-day all-cause mortality [HR 4.158, 95% CI 3.203–5.398 (low vs. normal BE) and HR 1.354, 95% CI 0.896–2.049 (high vs. normal BE)] and 90-day all-cause mortality [HR 4.078, 95% CI 3.160–5.263 (low vs. normal BE) and HR 1.369, 95% CI 0.917–2.045 (high vs. normal BE)], even after adjustment for significant prognostic covariates. The results were also consistent in subgroup analysis. RCS revealed an “L-type” relationship between BE and 28-day and 90-day all-cause mortality, as well as adjusting for confounding variables. Meanwhile, Kaplan–Meier survival curves were stratified by combining BE with carbon dioxide partial pressure (PaCO_2_), and patients had the highest mortality in the group which had low BE (< 3.5 mEq/L) and high PaCO_2_ (> 45 mmHg) compared with other groups.

**Conclusion:**

Our study revealed that low BE was significantly associated with 28-day and 90-day mortality in patients with AMI and indicated the value of stratifying the mortality risk of patients with AMI by BE.

## Introduction

The most common cause of cardiovascular disease (CVD) mortality worldwide is acute myocardial infarction (AMI), which is characterized by myocardial cell death caused by prolonged ischemia. More than 2.4 million people in the USA die annually from it, more than 4 million people in Europe and Northern Asia, and about one-third of people in developed countries die from it (1). Although the mortality of AMI has declined substantially in recent decades due to lifestyle and coronary revascularization, global health is substantially impacted by myocardial infarction (2), and complications after myocardial infarction are still a threat to human health. Therefore, it is very important to continue to find effective biological indicators that can predict patient outcomes, such as the risk of early mortality.

The arterial blood gas (ABG) test is a commonly ordered test in intensive care units (ICUs) that also analyses pH and blood gases in addition to electrolytes (3). According to the current European heart failure guidelines, patients with acute heart failure (AHF) should measure their blood pH, partial pressure of carbon dioxide (PaCO_2_), and lactate levels (4). Among patients with AMI, HF is the most powerful predictor of death and it has important implications for treatment (5). Thus, several studies explored the predictive value of the index of ABG in AMI and revealed that many indexes of ABG had great sensitivity in predicting the mortality of patients with AMI (6–8). For example, Xu et al. found that the higher anion gap (AG) was significantly associated with an increased risk of mortality, and the measure of AG can be a robust and reliable predictor of AMI mortality during follow-up (7). In a recent study, Zhang et al. concluded that acidemia may increase the 30-day survival rate and 90-day survival rate of patients with AMI admitted to the ICU, while acidemia is typically diagnosed based on the ABG with pH < 7.35 (9). However, Miñana et al. found that arterial PaO_2_, PaCO_2_, and pH did not correlate with all-cause long-term mortality in patients diagnosed with AHF (10). It is well known that pH is affected by both respiration and metabolic factors, and it may not fully reflect the true acid-base status. However, base excess (BE) represents an increase or decrease of alkali reserves in plasma, independent of respiratory factors.

In previous research, it was found that high BE, but not low BE, was an independent predictor of long-term mortality in patients with AHF (11), indicating that BE was important for assessing acid-base balance, especially in the context of stratifying the mortality risk in patients with AHF. However, It is unknown whether BE is related to mortality in patients with AMI hospitalized in the hospital. Accordingly, we aimed to understand whether BE may serve as a predictive value for a prognosis for patients with AMI.

## Materials and methods

### Source of data

Our study is a retrospective analysis, in which data are extracted from a vast critical care database named Medical Information Mart for Intensive Care III (MIMIC III). The MIMIC III database is a free and large public database that comprises de-identified and definitional health-related data, which contains over 40,000 patients admitted to intensive care units (ICU) of the Beth Israel Deaconess Medical Center (Boston, MA, United States) between 2001 and 2012 (12). We extracted data from MIMIC III after passing the National Institutes of Health (NIH) web-based training course for protected human study participants. We also passed the Collaborative Institutional Training Initiative examination and applied for data access. The ethical committee waived informed consent because the patients were de-identified, and the MIMIC database contained no protected information. To protect the privacy of the participants, their identification information was concealed.

### Participants

Patients with AMI who were admitted for the first time were included in our study. A total of 3,177 patients with AMI were identified using the International Classification of Diseases, Ninth Revision (ICD-9) codes. There were 192 patients who were under 18 years old or with an incorrect age and 520 patients without the result of BE. Thus, a total of 2,465 patients with AMI were ultimately enrolled in our study.

### Variables

All variables were extracted from the MIMIC III database using Structured Query Language (SQL) with PostgreSQL (version 9.6). The variables in our study included (1) physical characters, including age, gender, and body mass index (BMI); (2) types of hospital admission, including elective, emergency, and urgent; (3) past history, including hypertension, diabetes, dyslipidemia, atrial fibrillation (AF), acute kidney injury (AKI), chronic obstructive pulmonary disease (COPD), congestive heart failure (CHF), acute respiratory distress syndrome (ARDS), and sepsis; (4) vital signs, including systolic blood pressure (SBP), diastolic blood pressure (DBP), mean blood pressure (MBP), heart rate (HR), respiratory rate (RR), and temperature (T); (5) laboratory data, including pH, SpO_2_, PaCO_2_, bicarbonate (HCO_3_^–^), BE, AG, lactate, hemoglobin, platelet (PLT), white blood cell (WBC), albumin (ALB), urea nitrogen (BUN), creatinine (Scr), glucose, sodium, potassium, percutaneous coronary intervention (PCI), percutaneous transluminal coronary angioplasty (PTCA), coronary artery bypass grafting (CABG), Simplified Acute Physiology Score II (SAPS II), and Sequential Organ Failure Assessment (SOFA) score; and (6) oral medication, including aspirin, clopidogrel, beta blockers, diuretics, digitalis, and statin. All blood biochemical variables were the first measurement after the patient’s admission to the hospital before treatment.

### Clinical outcomes

Records from the Social Security Death Index provided information on survivorship (including survival outcome and death date). Notably, 28-day and 90-day all-cause mortality after the date of ICU admission were analyzed in our study.

### Statistical analysis

Variables in the categorical form are presented as numbers (percentages) and variables in continuous form as mean ± standard deviation if the distribution is normally distributed and median (interquartile range) otherwise. Continuous variables were analyzed using analysis of variance or Kruskal-Wallis tests to determine baseline differences among groups stratified by BE, and the chi-squared test or Fisher’s exact test was used for categorical variables. We also examined the relationship between BE and the outcomes (with the normal BE group as the reference group) using both univariate and multivariate Cox proportional-hazards models. Variables with *P* < 0.05 in the univariate Cox proportional-hazards model were entered into separate multivariate models for 28-day and 90-day all-cause mortality: model 1, crude; model 2, included age, gender, SBP, and DBP; model 3, included variables in model 2 and hypertension, AF, COPD, AKI, sepsis, and CHF; model 4, included variables in model 3 and aspirin, clopidogrel, beta-blockers, diuretics, digitalis, statin, PCI, and CABG; model 5, included variables in model 4 and sodium, potassium, ALB, BUN, Scr, AG, and SpO_2_. Meanwhile, restricted cubic splines (RCS) were used to separately explore the relationship between BE and hazard ratio (HR) of 28-day and 90-day mortality. The cumulative incidence of 28-day and 90-day all-cause mortality was presented using the Kaplan-Meier curve. Additionally, Kaplan-Meier curves were also generated for BE combined with PaCO_2_ as the respiratory factor. BE cutoff values (–3.5 mEq/L) were determined by receiver operating characteristic (ROC) analysis using the Youden index for 28-day all-cause mortality. To explore the link between admission BE values (modeled as continuous variables) and the risk of 28 and 90-day all-cause mortality, RCS was used. All analyses were performed using R (R Foundation for Statistical Computing, Vienna, Austria). All tests were two-sided, and *P* < 0.05 was considered statistically significant.

## Results

### Baseline characters

In total, 2,465 patients with AMI were included in our study, among which 1,430 (58.0%), 677 (27.5%), and 358 (14.5%) patients were classified as having normal BE, low BE, and high BE, respectively. The median age was 67 years and 1647 (67.9%) patients were male. Baseline clinical characteristics of patients stratified by BE are shown in Table 1. Patents with low BE were younger with an average age of 68.23. Meanwhile, patients with low BE were more likely to have higher HR, RR, AG, hemoglobin, WBC, BUN, Scr, glucose, and potassium. Moreover, as BE declined, the rate of AKI, ARDS, sepsis, SAPS II score, PCI, CABG, 28-day mortality, 90-day mortality, cardiac arrest, and cardiac shock was increasing. However, patients with low BE had lower SBP, DBP, MBP, T, pH, SpO_2_, PaCO_2_, HCO_3_^–^, PLT, and ALB as well as the rate of hypertension, diabetes, and invasive ventilation.

**TABLE 1:** Baseline characteristics across base excess values.

Variable	Overall	Low BE (BE < –2.0)	Normal BE (–2.0 ≤ BE ≤ 2.0)	High BE (BE > 2.0)	*P*-value
N (%)	2,465	677 (27.5)	1,430 (58.0)	358 (14.5)	<0.001
Age, years	67.23 ± 12.22	68.23 ± 12.57	66.25 ± 12.27	69.25 ± 10.88	<0.001
Male, n (%)	1,674 (67.9)	422 (62.3)	1,016 (71.0)	236 (65.9)	<0.001
BMI, kg/m^2^	28.31 ± 3.58	29.97 ± 15.19	29.01 ± 5.86	28.96 ± 13.05	0.074
**Types of hospital admission, n (%)**					
Elective	525 (21.3)	103 (15.2)	341 (23.8)	81 (22.6)	<0.001
Emergency	1,795 (72.8)	529 (78.1)	1,003 (70.1)	263 (73.5)	
Urgent	145 (6.0)	45 (6.6)	86 (6.0)	14 (3.9)	
**Past history, n (%)**					
AF	808 (32.8)	220 (32.5)	444 (31.0)	144 (40.2)	0.004
Hypertention	1,332 (54.0)	303 (44.8)	832 (58.2)	197 (55.0)	<0.001
Diabetes	804 (32.6)	211 (31.2)	462 (32.3)	131 (36.6)	0.194
Dyslipidemia	557 (22.6)	133 (19.6)	344 (24.1)	80 (22.3)	0.077
COPD	32 (1.3)	7 (1.0)	13 (0.9)	12 (3.4)	0.001
AKI	395 (16.0)	173 (25.6)	171 (12.0)	51 (14.2)	<0.001
ARDS	129 (5.2)	47 (6.9)	63 (4.4)	19 (5.3)	0.051
Sepsis	86 (3.5)	42 (6.2)	33 (2.3)	11 (3.1)	<0.001
CHF	781 (31.7)	240 (35.5)	392 (27.4)	149 (41.6)	<0.001
**Vital signs on admission**					
SBP, mmHg	112.99 ± 14.39	109.64 ± 15.67	114.06 ± 13.42	114.96 ± 14.66	<0.001
DBP, mmHg	58.51 ± 8.98	57.78 ± 9,55	58.97 ± 8,82	58.04 ± 8.43	0.011
MBP, mmHg	76.33 ± 9.28	75.04 ± 10.37	77.01 ± 8.77	76.03 ± 8,87	<0.001
HR, beats/min	83.52 ± 13.68	87.41 ± 15.44	83.03 ± 12.68	83.79 ± 13.95	0.092
RR, times/min	18.22 ± 3.57	18.75 ± 3.92	17.99 ± 3.34	18.21 ± 3.69	<0.001
T,°C	36.84 ± 0.63	36.76 ± 0.78	36.88 ± 0.56	36.83 ± 0.60	<0.001
**Laboratory data on admission**					
pH	7.38 ± 0.08	7.34 ± 0.10	7.39 ± 0.06	7.41 ± 0.07	<0.001
SpO_2_,%	97.44 ± 2.63	96.90 ± 4.13	97.68 ± 1.71	97.49 ± 1,81	<0.001
PaCO_2_, mmHg	41.97 ± 9.80	40.42 ± 10.90	42.03 ± 8.12	44.81 ± 9.796	<0.001
HCO3^–^, mmol/L	23.65 ± 5.57	20.87 ± 5.40	24.10 ± 4.57	27.59 ± 5.73	<0.001
BE, mmol/L	–0.99 ± 4.49	–6.28 ± 3.96	–0.03 ± 1.13	5.20 ± 3.04	<0.001
AG, mmol/L	13.73 ± 3.47	14.80 ± 4.30	13.32 ± 2.87	13.34 ± 3.54	<0.001
Lactate, mmol/L	1.80 (1.2,2.6)	1.9 (1.3,3.2)	1.7 (1.2,2.4)	1.6 (1.2,2.5)	<0.001
Hemoglobin, g/dL	10.99 ± 1.98	11.09 ± 1.99	10.98 ± 1.99	10.81 ± 1.89	0.084
PLT, K/μL	226.42 ± 112.81	222.80 ± 120.87	226.47 ± 109.25	233.03 ± 111.14	0.382
WBC, K/μL	11.03 ± 5.83	11.78 ± 6.38	10.73 ± 5.86	10.77 ± 4.39	<0.001
ALB, g/dL	3.45 ± 0.71	3.24 ± 0.76	3.55 ± 0.68	3.44 ± 0.65	<0.001
BUN, mg/dL	7.23 ± 3.45	30.14 ± 23.53	22.93 ± 16.41	26.52 ± 19.54	<0.001
Scr (mg/dL)	1.33 ± 1.21	1.59 ± 1.46	1.20 ± 0.97	1.36 ± 1.25	<0.001
Glucose (g/dL)	140.03 ± 60.33	150.97 ± 76.65	134.92 ± 49.26	139.73 ± 62.82	<0.001
Sodium, mEq/L	138.20 ± 2.86	138.32 ± 3.22	138.12 ± 2.65	138.31 ± 2.92	0.242
Potassium, mEq/L	4.21 ± 0.62	4.27 ± 0.66	4.19 ± 0.57	4.17 ± 0.73	0.004
**Oral medication on admission**					
Aspirin, n (%)	2,045 (83.0)	534 (78.9)	1,202 (84.1)	309 (86.3)	0.002
Clopidogrel, n (%)	1,110 (45.0)	330 (48.7)	635 (44.4)	145 (40.5)	0.031
Beta blockers, n (%)	1,846 (74.9)	437 (64.5)	1,127 (78.8)	282 (78.8)	<0.001
Diuretics, n (%)	1,572 (63.8)	366 (54.1)	950 (66.4)	256 (71.5)	<0.001
Digitalis, n (%)	111 (4.5)	31 (4.6)	50 (3.5)	30 (8.4)	<0.001
Statin, n (%)	1,761 (71.4)	445 (65.7)	1,051 (73.5)	265 (74.0)	<0.001
PCI, n (%)	753 (30.5)	306 (35.5)	362 (29.1)	85 (23.7)	<0.001
PTCA, n (%)	330 (13.4)	117 (13.6)	163 (13.1)	50 (14.0)	0.089
CABG, n (%)	1,007 (40.9)	260 (30.2)	606 (48.6)	141 (39.4)	<0.001
SOFA	4 (2.6)	4 (2.8)	4 (2.5)	4 (2.6)	<0.001
SAPSII	33 (26.43)	37 (28.51)	31 (25.39)	34 (27.42)	<0.001
**Clinical outcome, n (%)**					
14-day mortality	249 (10.1)	151 (22.3)	73 (5.1)	25 (7.0)	<0.001
28-day mortality	280 (11.4)	159 (23.5)	90 (6.3)	31 (8.7)	<0.001
90-day mortality	292 (11.8)	164 (24.2)	95 (6.6)	33 (9.2)	<0.001
Cardiac arrest	167 (6.8)	80 (11.8)	64 (4.5)	23 (6.4)	<0.001
Cardiac shock	314 (12.7)	142 (21.0)	129 (9.0)	43 (12.0)	<0.001
Non-invasive ventilation	65 (2.6)	15 (2.2)	30 (2.1)	20 (5.6)	0.001
Invasive ventilation	590 (23.9)	143 (21.1)	350 (24.5)	97 (27.1)	0.077

Continuous variables are presented as mean ± SD if normally distributed, and median (interquartile range) if not normally distributed. Categorical variables are presented as number of patients (%).

BMI, body mass index; AF, atrial fibrillation; COPD, chronic obstructive pulmonary disease; AKI, acute kidney injury; ARDS, CHF, congestive heart failure; SBP, systolic blood pressure; DBP, diastolic blood pressure; MBP, mean blood pressure; HR, heart rate; RR, respiratory rate; HCO3^–^, bicarbonate; PaCO_2_, carbon dioxide partial pressure; PaO_2_, partial pressure of arterial oxygen; BE, base excess; AG, anion gap; PLT, platelet; WBC, white blood cell; ALB, albumin; BUN, blood urea nitrogen; Scr, serum creatinine; PCI, percutaneous coronary intervention; PTCA, Percutaneous transluminal coronary angioplasty; CABG, coronary artery bypass grafting; SOFA, sequential organ failure assessment; SAPS II, simplified acute physiology score.

### The association between base excess and the mortality of acute myocardial infarction

The unadjusted Cox proportional hazard regression model showed that the low BE group, but not a high BE group, was an independent determinant of the risk of 28-day mortality (HR 4.158, 95% CI 3.203–5.398) (Table 2). Despite further adjustments, the associations remained significant with a 1.595-fold increased risk (95% CI 1.153–2.206) in the final model. Meanwhile, the low BE group was still an independent determinant of the risk of 90-day mortality (HR 4.078, 95% CI 3.160–5.263), and the associations were still significant in the model after adjusting the relevant factors (Table 3).

**TABLE 2:** Cox proportional hazard models for 28-day all-cause death.

Variables	Normal BE (–2.0 ≤ BE ≤ 2.0)	Low BE (BE < –2.0)	High BE (BE > 2.0)
Model 1^a^	1.000 (Ref.)	4.158 (3.203–5.398)	1.354 (0.896–2.049)
*P*-value	–	<0.001	0.151
Model 2^b^	1.000 (Ref.)	3.288 (2.515–4.299)	1.147 (0.749–1.757)
*P*-value	–	<0.001	0.527
Model 3^c^	1.000 (Ref.)	2.641 (2.012–3.465)	1.100 (0.718–1.684)
*P*-value	–	<0.001	0.662
Model 4^d^	1.000 (Ref.)	2.115 (1.606–2.787)	0.989 (0.645–1.518)
*P*-value	–	<0.001	0.961
Model 5^e^	1.000 (Ref.)	1.595 (1.153–2.206)	1.012 (0.643–1.595)
*P*-value	–	0.005	0.957

^a^Model 1 Univariate model.

^b^Model 2 adjusted for age, gender, SBP, DBP.

^c^Model 3 adjusted for model 2 plus hypertension, AF, COPD, AKI, SEPSIS, CHF.

^d^Model 4 adjusted for model 3 plus Aspirin, Clopidogrel, Beta blockers, Diuretics, Digitalis, Statin, PCI, CABG.

^e^Model 5 adjusted for model 4 plus Sodium, Potassium, ALB, BUN, Scr, AG, SpO_2_.

**TABLE 3:** Cox proportional hazard models for 90-day all-cause death.

Variables	Normal BE (–2.0 ≤ BE ≤ 2.0)	Low BE (BE < –2.0)	High BE (BE > 2.0)
Model 1^a^	1.000 (Ref.)	4.078 (3.160–5.263)	1.369 (0.917–2.045)
*P*-value	–	<0.001	0.125
Model 2^b^	1.000 (Ref.)	3.240 (2.494–4.209)	1.164 (0.771–1.758)
*P*-value	–	<0.001	0.469
Model 3^c^	1.000 (Ref.)	2.565 (1.967–3.344)	1.115 (0.738–1.684)
*P*-value	–	<0.001	0.605
Model 4^d^	1.000 (Ref.)	2.081 (1.590–2.723)	1.105 (0.671–1.536)
*P*-value	–	<0.001	0.943
Model 5^e^	1.000 (Ref.)	1.556 (1.136–2.130)	1.038 (0.670–1.608)
*P*-value	–	0.006	0.867

^a^Model 1 Univariate model.

^b^Model 2 adjusted for age, gender, SBP, DBP.

^c^Model 3 adjusted for model 2 plus hypertension, AF, COPD, AKI, SEPSIS, CHF.

^d^Model 4 adjusted for model 3 plus Aspirin, Clopidogrel, Beta blockers, Diuretics, Digitalis, Statin, PCI, CABG.

^e^Model 5 adjusted for model 4 plus Sodium, Potassium, ALB, BUN, Scr, AG, SpO_2_.

**FIGURE 1:**
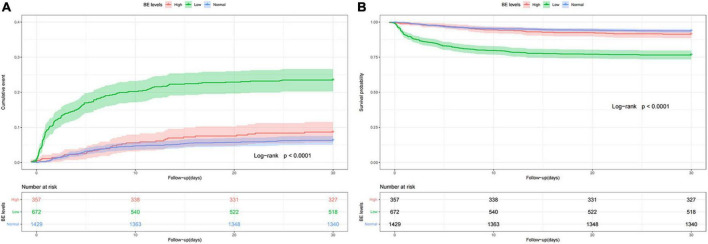
Cumulative incidence **(A)** and Kaplan-Meier curve **(B)** of 28-day all-cause mortality stratified by base excess.

**FIGURE 2:**
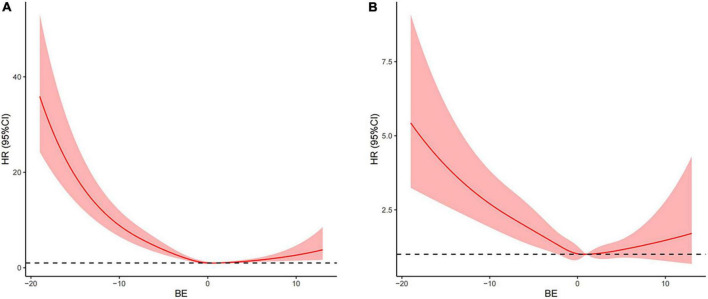
Associations between base excess (BE) on a continuous scale and adjusted risk of 28-day all-cause mortality in patients with AMI. Crude hazard ratio (HR) and 95% CI for BE in 28-day mortality **(A)**. Adjusted HR and 95% CI for BE in 28-day mortality **(B)**. The analyses used a model with restricted cubic splines. Adjusted variables included age, gender, systolic blood pressure (SBP), diastolic blood pressure (DBP), hypertension, atrial fibrillation (AF), chronic obstructive pulmonary disease (COPD), acute kidney injury (AKI), sepsis, congestive heart failure (CHF) aspirin, clopidogrel, beta-blockers, diuretics, digitalis, statin, percutaneous coronary intervention (PCI), coronary artery bypass grafting (CABG), sodium, potassium, albumin (ALB), urea nitrogen (BUN), creatinine (Scr), anion gap (AG), and SpO_2_, namely, model 5 described above.

To further explore the relationship between the BE and mortality of AMI, an analysis of Kaplan-Meier data revealed that the low BE group had significantly higher 28-day mortality (Figure 1) and 90-day mortality (Supplementary Figure 1) compared with the normal and high BE groups. Based on admission BE estimates, the RCS corresponding to the risk of 28-day all-cause mortality showed an “L-type” relationship between BE and the risk of mortality (Figure 2A). After adjustment by confounders including gender, age, SBP, DBP, hypertension, AF, COPD, AKI, sepsis, CHF, sodium, potassium, ALB, BUN, Scr, AG, and SpO_2_, as well as the use of aspirin, clopidogrel, beta-blockers, diuretics, digitalis, statin, PCI, and CABG, there was still an “L-type” relationship between BE and risk of 28-day all-cause mortality (Figure 2B). Similarly, the restricted spline curve also revealed that an “L-type” relationship between BE and 90-day mortality (Supplementary Figure 2A) and “L-type” relationship still exist after adjusting confounders (Supplementary Figure 2B).

### Subgroup analysis

**FIGURE 3:**
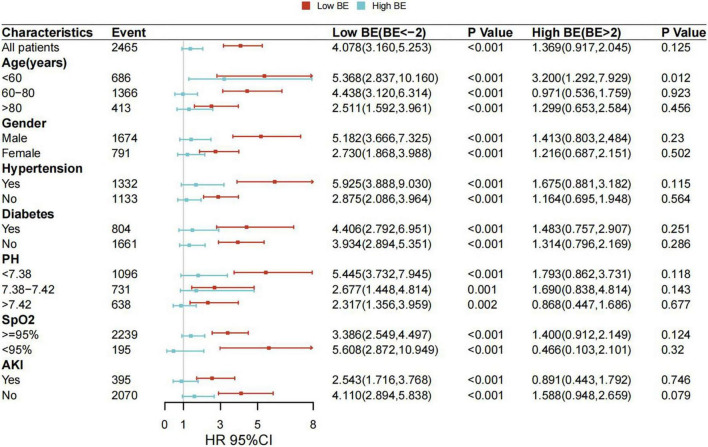
Associations between BE and risk of all-cause mortality in subgroups. Forest plot and adjusted HRs with 95% CI for 28-day all-cause mortality.

To further investigate whether the relationship would be different in various conditions, subgroup analyses were conducted for age, gender, hypertension, diabetes, pH, SpO_2_, and AKI, and the correlations between low BE and mortality in patients with AMI remained statistically significant in patients with different subgroups. After adjustment for the confounders, the HRs of 28-day mortality were generally increased in patients in the low BE group (Figure 3).

### Relationship between base excess combined with carbon dioxide partial pressure and acute myocardial infarction mortality

**FIGURE 4:**
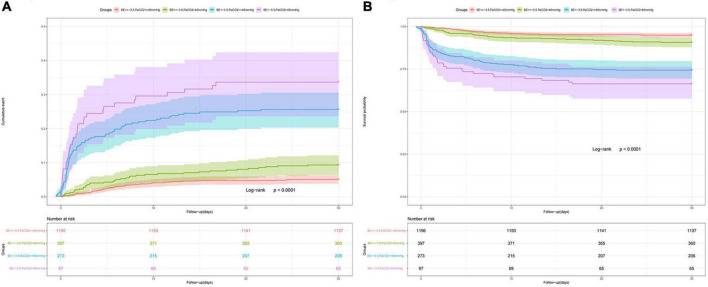
Cumulative incidence **(A)** and Kaplan-Meier curve **(B)** of 28-day all-cause mortality stratified by BE and PaCO_2_. BE cutoff values (–3.5 mEq/L) were determined by receiver operating characteristic (ROC) analysis for 28-day all-cause mortality using the Youden index.

To further determine the relationship between BE and mortality, we added PaCO_2_ as a respiratory component to BE. BE cutoff values (–3.5 mEq/L) were determined by ROC analysis for 28-day all-cause mortality using the Youden index (Supplementary Figure 3). Kaplan–Meier survival curves stratified by a combination of admission BE and PaCO_2_ are shown in Figure 4, which revealed that low BE with high PaCO_2_ level was associated with the highest 28-day all-cause mortality (Figure 4) and the highest 90-day all-cause mortality (Supplementary Figure 4) among the groups.

## Discussion

Our retrospective study of 2,456 patients with AMI showed that low BE was significantly associated with increased 28-day and 90-day all-cause mortality. The correlations between low BE levels and risk of cardiovascular events were independent of other cardiovascular risk factors, and they remained significantly stable in subgroup analyses. In contrast, high BE had no correlation to the mortality of patients with AMI. Moreover, an “L-type” association between BE and 28-day and 90-day all-cause mortality was found. These results indicated meaningful predictive implications in the clinical practice of BE.

Several studies have shown that BE is one of the most important tools for determining the severity of illness in acute care settings (9, 10, 13, 14). Miñana et al. revealed that admission pH, arterial PaO_2_, and PaCO_2_ had no association with all-cause mortality in patients with HF (10). However, Park et al. found that the most popular acid-base imbalance was respiratory alkalosis in patients with high-risk AHF, and pH provided additional prognostic value for patients with high-risk AHF and may be helpful for risk stratification and patient care. They also reported that there was no relationship between higher mortality with alkalosis or neutral pH as well as the cause of acidosis had no association with mortality of patients with AHF (15). However, Tang Zhang et al. observed that severe acidemia (non-acidemia, 7.35 ≤ pH ≤ 7.45; mild acidemia, 7.25 ≤ pH ≤ 7.35; severe acidemia, pH < 7.25) could increase the 30-day mortality in patients with AMI (9). Our findings were partly consistent with the results of Tang Zhang et al. and we found that the 28-day and 90-day all-cause mortality was closely related to acidemia in patients with AMI only when BE was low. Meanwhile, we found that the risks of 28-day and 90-day mortality were elevated in patients with AMI when BE was high regardless of whether the pH was acidic, neutral, or alkaline, and after adjusting for potential risk factors and covariates, the association remains significant. Therefore, BE could be a convenient and intuitive index for prognoses of patients with AMI independent of conventional cardiovascular risk factors.

There are several factors affecting BE concentrations and many possible reasons responsible for the difference between our results and others. Previous literature showed that the acid-base balance was first identified by pH. Nevertheless, pH was affected by metabolic and respiratory status. To stratify risk based on alkalemia or acidemia, only patients with those conditions were further investigated (16). Meanwhile, although the pH appeared to be neutral, it might be caused by mixed acid-base disturbances and might mask the actual existence of acid-base imbalance. In addition, some drugs and diseases could also affect the acid-base balance. For example, patients taking diuretics or who had chronic kidney disease or COPD frequently suffered from metabolic alkalosis, metabolic acidosis, or respiratory acidosis. At the same time, BE was considered to be the first accurate measurement of acid-base balance derived from non-respiratory sources (17). Thus, we first stratified the mortality risk by BE, which could separate respiratory factors from acid-base balance, revealing that low BE had the worst outcome of 28-day and 90-day mortality compared with high BE and normal BE. Meanwhile, RCS also showed that BE tended to associate with a worse prognosis with the “L-type” relationship in unadjusted models, and there also existed an “L-type” relationship after adjusting by covariates. Our findings indicated that the short-term prognosis in patients with AMI with metabolic acidosis, as determined by BE level at admission, is significantly worse than that of patients with metabolic alkalosis.

Metabolic acidosis is divided into high AG metabolic acidosis and hyperchloremic or normal AG metabolic acidosis. In addition to lactic acidosis and ketoacidosis, metabolic acidosis could be caused by ethylene glycol, methanol, and salicylate intoxication. Similarly, hyperchloremic metabolic acidosis is most commonly caused by renal tubular acidosis, gastrointestinal bicarbonate depletion, drug-induced hyperkalemia, early renal failure, and acid infusion. In patients with AMI, falling cardiac output combined with arterial hypoxemia causes tissue hypoxia, metabolic acidosis, and a drop in plasma bicarbonate due to lactic acid accumulation. Acidosis of the metabolism increases with disease severity and is an important cause of death. The build-up of acidic metabolites during cardiac ischemia results in a drop in intracellular and extracellular pH, reaching as low as 6.0–6.5. Ischemic injury is exacerbated by resulting tissue acidosis, which negatively affects cardiac function (18). In an earlier study of 50 patients with AMI, serum pH < 7.35 was significantly correlated with a patient mortality rate of > 60% (19). Bicarbonate can also reflect an acid-base state. In epidemiological studies, lower bicarbonate levels are associated with hypertension, both prevalent and incident (20–22), insulin resistance (23), progression of kidney disease (24), and mortality (25). These findings from previous literature show that metabolic acidosis worsens the prognosis supporting our results. But a cohort study of 6,229 community adults showed opposite results, compared with bicarbonate 23–24 mEq/L, and bicarbonate ≥ 25 mEq/L was associated with 3.0 g of greater LVM (95% CI 0.5–5.0) and 1.0 mm Hg of higher aortic PP (95% CI 0.4–2.0). Increases in bicarbonate concentration were associated with a 13% greater risk of HF (HR 1.13, 95% CI 1.01–2.11) (26), showing that higher serum bicarbonate levels were related to CVD subclinical stages and new HF. Additionally, among a well-characterized but smaller group of diabetics in Australia, higher serum bicarbonate was associated with a lower risk of incident coronary heart disease, but not HF (27). Evidently, although there are few studies that link hypercapnia with AMI, it is crucial to gain a better understanding of how acid-base balance impacts cardiovascular function.

Furthermore, our study revealed that the patients with coexisting metabolic acidosis and respiratory acidosis could have the worst prognosis among the four groups which had different BE levels and PaCO_2_. Hypercapnia can often be found in patients with AMI requiring intubation, and it is always associated with severe lung diseases or heart disease (28, 29). Notably, BE was a significant independent determinant of 28-day and 90-day all-cause mortality, but PaCO_2_ was not an independent variable in our study. When we combined the BE with PaCO_2_ to explore the prognosis of patients with AMI, the predictive efficiency would be much better. Therefore, our results could indicate that PaCO_2_ would be useful for risk stratification when in conjunction with BE.

## Conclusion

Our study revealed that low BE was significantly associated with 28 and 90-day mortality in patients with AMI and indicated the value of stratifying the mortality risk of patients with AMI by BE, especially when combined with PaCO_2_. BE, as an easily obtained and important marker on admission for critically ill patients, could be used as a reliable predictor of prognosis in patients with AMI.

### Limitations

Our study has some limitations. First, our study is an observational study, and we cannot determine the causal relationship between BE and the mortality of patients with AMI, so there needs a cohort study to explore the mechanism. Second, AMI-related risk factors were included as much as possible in our study, such as comorbidities and laboratory examinations. However, due to the limitations of the data, other residual confounding risk factors may not be included in the logistic model. So more detailed clinical cohort studies are still needed to support our study conclusions. Third, this study was limited to short-term outcomes, and data on the relationship between BE level and long-term outcomes of patients with AMI are still missing.

## Data availability statement

Publicly available datasets were analyzed in this study. This data can be found here: https://mimic.mit.edu/docs/gettingstarted/.

## Author contributions

CL, ZD, and GT designed the study. CL, ZD, TZ, QL, and DH analyzed and interpreted the data. CL and ZD drafted the manuscript. CL, ZD, TZ, DW, BW, PG, and GT revised the manuscript. All authors gave final approval of the final version to be published.

## Supplementary material

Supplementary Figure 1 Cumulative incidence **(A)** and Kaplan-Meier curve **(B)** of 90-day all-cause mortality stratified by base excess.

Supplementary Figure 2 Associations between base excess (BE) on a continuous scale and adjusted risk of 90-day all-cause mortality in patients with AMI. Crude hazard ratio (HR) and 95% CI for BE in 90-day mortality **(A)**. Adjusted HR and 95% CI for BE in 90-day mortality **(B)**. The analyses used a model with restricted cubic splines. Adjusted variables included age, gender, systolic blood pressure (SBP), diastolic blood pressure (DBP), hypertension, atrial fibrillation (AF), chronic obstructive pulmonary disease (COPD), acute kidney injury (AKI), sepsis, congestive heart failure (CHF) aspirin, clopidogrel, beta-blockers, diuretics, digitalis, statin, percutaneous coronary intervention (PCI), coronary artery bypass grafting (CABG), sodium, potassium, albumin (ALB), urea nitrogen (BUN), creatinine (Scr), anion gap (AG), and SpO_2_, namely, model 5 described above.

Supplementary Figure 3 Receiver operating characteristic (ROC) analysis for the diagnostic accuracy of BE in 28-day all-cause mortality.

Supplementary Figure 4 Cumulative incidence **(A)** and Kaplan-Meier curve **(B)** of 90-day all-cause mortality stratified by BE and PaCO_2_.
