# Supplementary figures and images for: Base excess is associated with the risk of all-cause mortality in critically ill patients with acute myocardial infarction
Source: Front Cardiovasc Med. 2022 Aug 9;9:942485. doi: 10.3389/fcvm.2022.942485 (PMC9396255; doi:10.3389/fcvm.2022.942485)

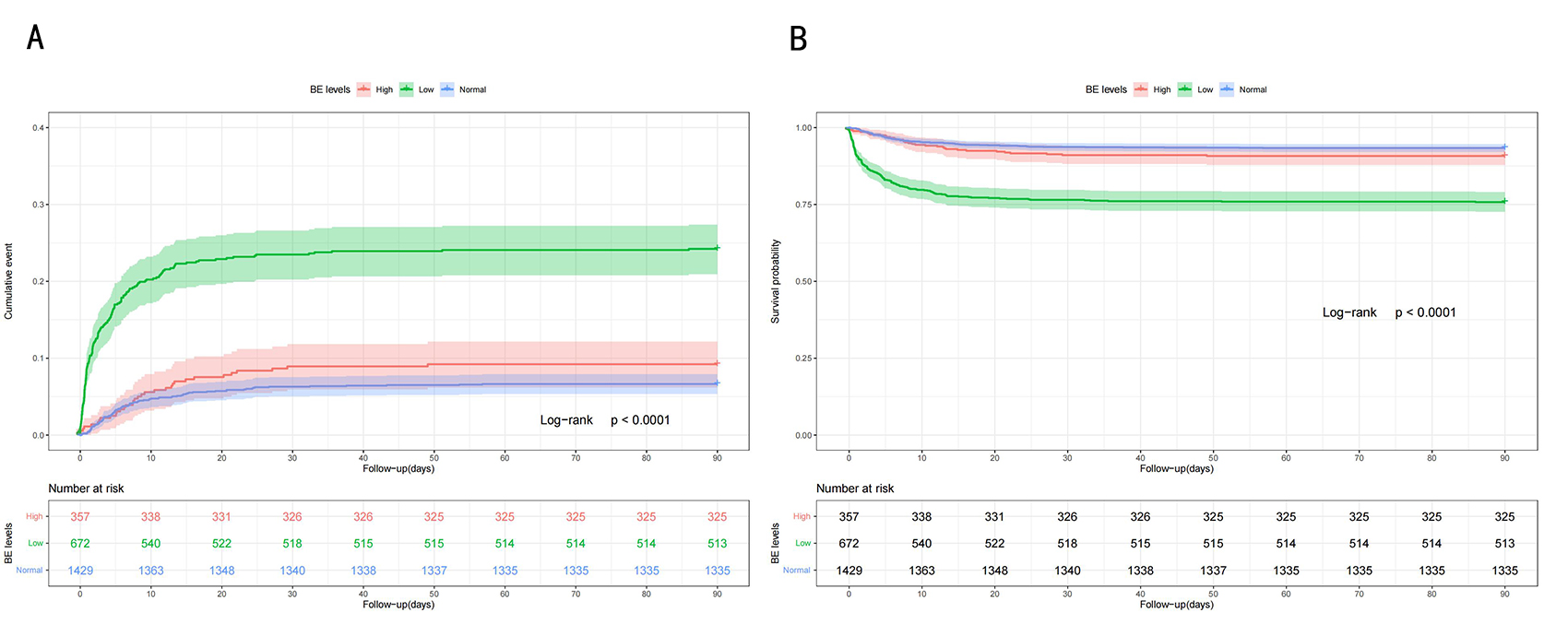

Supplement: Supplementary Figure 1 — Cumulative incidence (A) and Kaplan-Meier curve (B) of 90-day all-cause mortality stratified by base excess. [file Image_1.JPEG]

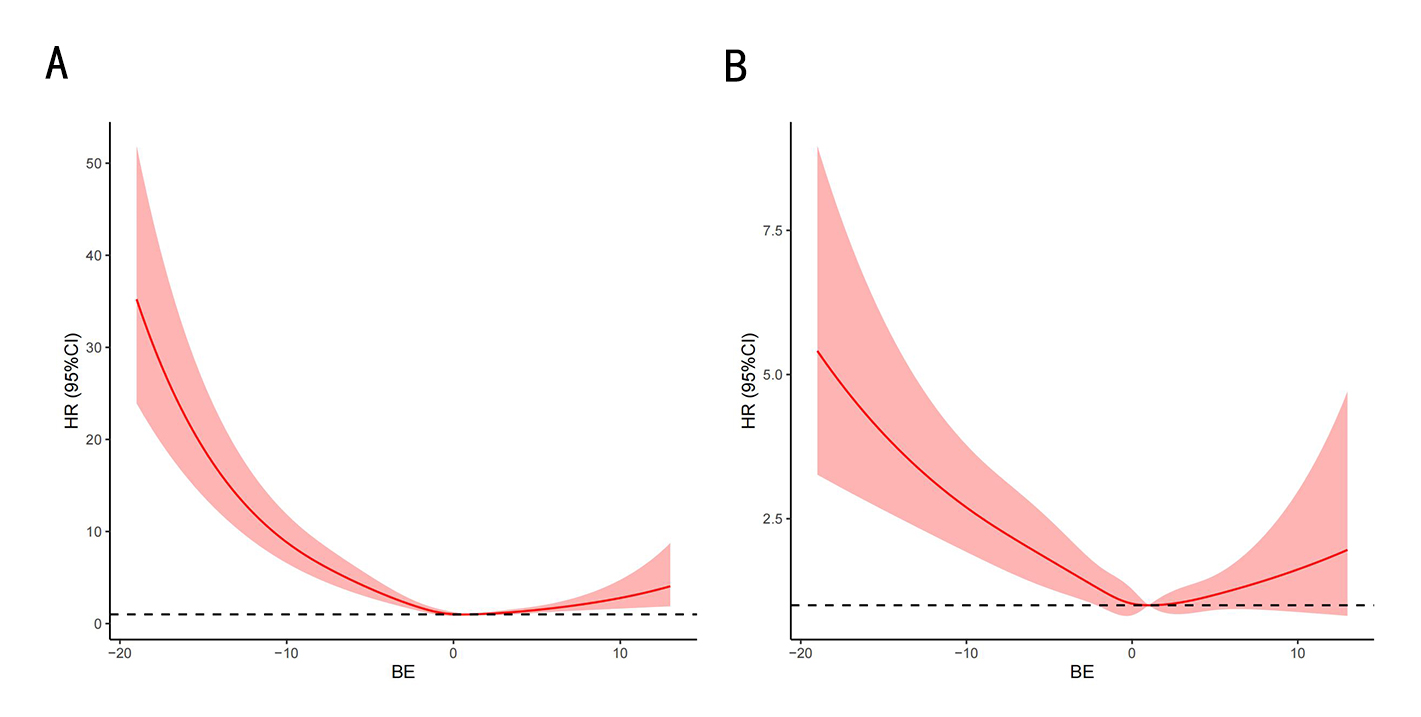

Supplement: Supplementary Figure 2 — Associations between base excess (BE) on a continuous scale and adjusted risk of 90-day all-cause mortality in patients with AMI. Crude hazard ratio (HR) and 95% CI for BE in 90-day mortality (A). Adjusted HR and 95% CI for BE in 90-day mortality (B). The analyses used a model with restricted cubic splines. Adjusted variables included age, gender, systolic blood pressure (SBP), diastolic blood pressure (DBP), hypertension, atrial fibrillation (AF), chronic obstructive pulmonary disease (COPD), acute kidney injury (AKI), sepsis, congestive heart failure (CHF) aspirin, clopidogrel, beta-blockers, diuretics, digitalis, statin, percutaneous coronary intervention (PCI), coronary artery bypass grafting (CABG), sodium, potassium, albumin (ALB), urea nitrogen (BUN), creatinine (Scr), anion gap (AG), and SpO2, namely, model 5 described above. [file Image_2.JPEG]

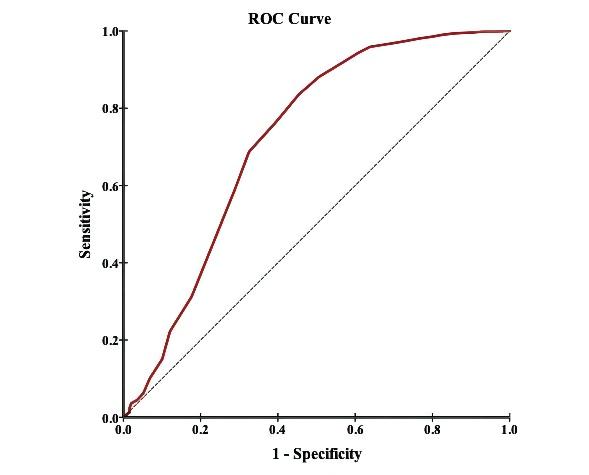

Supplement: Supplementary Figure 3 — Receiver operating characteristic (ROC) analysis for the diagnostic accuracy of BE in 28-day all-cause mortality. [file Image_3.JPEG]

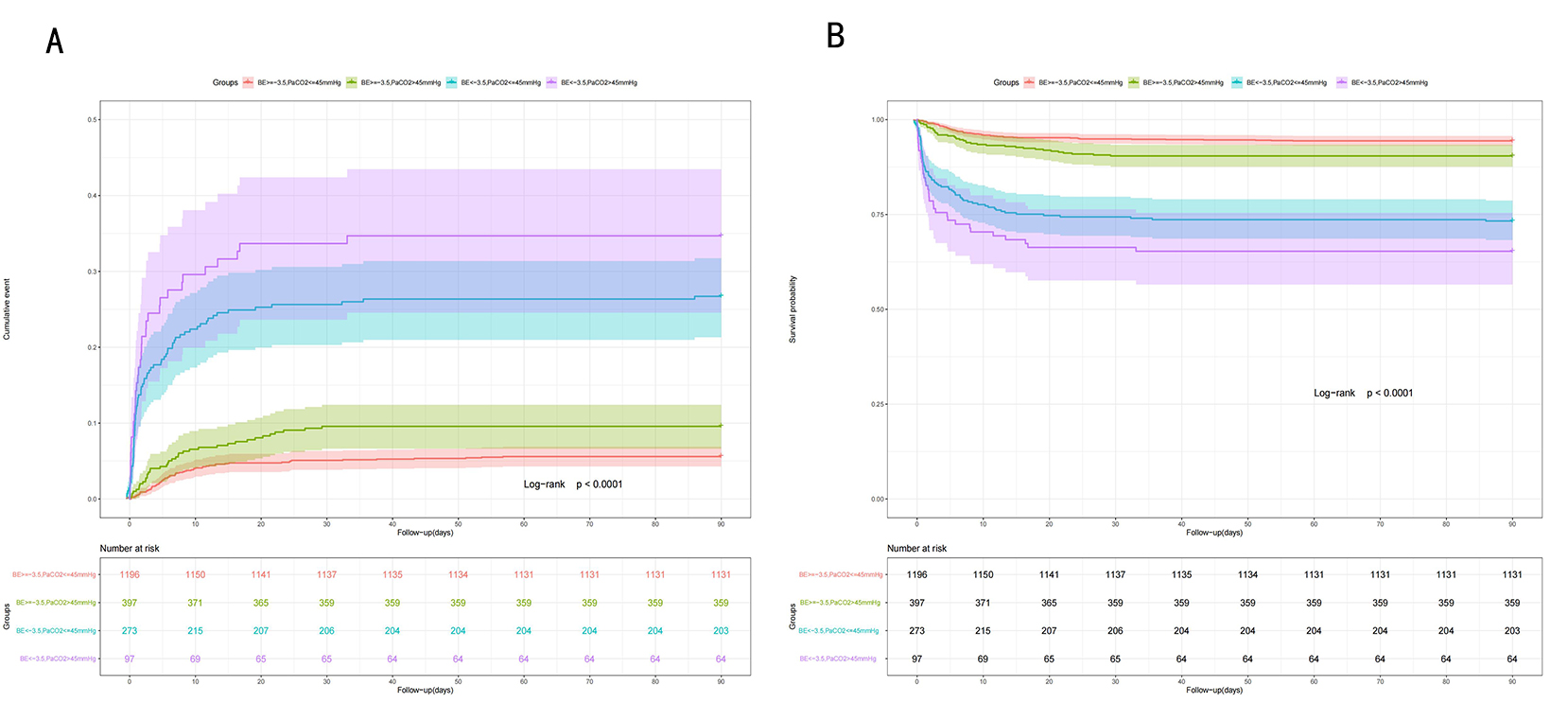

Supplement: Supplementary Figure 4 — Cumulative incidence (A) and Kaplan-Meier curve (B) of 90-day all-cause mortality stratified by BE and PaCO2. [file Image_4.JPEG]
